# 

**Published:** 1908-02-15

**Authors:** 


CONTENTS

| | | |
|---|---|---|
| Event and Comment | | 55 |
| Care of the Deciduous Teeth, | *By O. W. White, D.D.S,* | 58 |
| Cast Gold Inlays, | *By Edwin E. Harcourt, D.D.S.* | 82 |
| Vaseline, | *By W. G. Hamm, D.D.S.* | 86 |
| Pulp Reunion, | *By A. H. Fuller, M.D., D.D.S.* | 87 |
| Indents | | 90 |
| Announcements | | 105 |

